# Proliferation of Lung Epithelial Cells Is Regulated by the Mechanisms of Autophagy Upon Exposure of Soots

**DOI:** 10.3389/fcell.2021.662597

**Published:** 2021-07-21

**Authors:** Rituraj Niranjan, Kaushal Prasad Mishra, Sachchida Nand Tripathi, Ashwani Kumar Thakur

**Affiliations:** ^1^Laboratory 6, Department of Biological Sciences and Bioengineering (BSBE), Indian Institute of Technology, Kanpur, India; ^2^Talent Search Scientist (TSS-ICMR), currently at, Immunology Laboratory, ICMR-Vector Control Research Centre, Puducherry, India; ^3^Department of Civil Engineering, Centre for Environmental Science and Engineering, Indian Institute of Technology Kanpur, Kanpur, India

**Keywords:** p62/SQSTM1, reactive oxygen species, particulate matter, autophagy, fullerene soot, cell proliferation, environmental soots, pollutants (environmental)

## Abstract

**Background:**

Soots are known to cause many diseases in humans, but their underlying mechanisms of toxicity are still not known. Here, we report that soots induce cell proliferation of lung epithelial cells *via* modulating autophagy pathways.

**Results:**

Fullerene soot and diesel exhaust particles (DEP) induced cell proliferation of lung epithelial, A549 cells *via* distinct autophagic mechanisms and did not cause cell death. Exposure of fullerene soot protected the cell death of A549 cells, caused by hydrogen peroxide, and inhibited LPS-induced autophagy. Fullerene soot co-localized with the autophagic proteins and inhibited starvation-induced autophagy (downregulated ATG-5, beclin-1, p62, and LC3 expressions) independent of its antioxidant properties. Similarly, it decreased the expression profile of autophagic genes and upregulated the proliferation-responsive gene, Ki-67, in mice. We observed that expressions of fullerene soot-responsive genes (Beclin-1, ATG-5, and p62) were reverted by Akt Inhibitor X, indicating an important role of the Akt pathway. At an elemental level, we found that elemental carbon of fullerene soot may be converted into organic carbon, as measured by OCEC, which may point fullerene soot as a source of carbon. On the other hand, DEP upregulated the expressions of autophagy genes. Akt Inhibitor X did not attenuate DEP-induced cell proliferation and autophagic response. However, an autophagic inhibitor, chloroquine, and significantly inhibited DEP-induced cell proliferation.

**Conclusion:**

It can be said that distinct autophagic mechanisms are operational in cell proliferation of lung epithelial cells due to soots, which may be responsible for different diseases. Understanding the mechanism of these pathways provides some important targets, which can be utilized for the development of future therapeutics.

## Background

Ambient soot/black carbon- and traffic-related air pollution is responsible for the development of many diseases of humans and animals (Abas et al., 2004; Jacquemin et al., 2012; Hedman et al., 2015; Ji et al., 2015; Niranjan and Thakur, 2017). Soot consists of carbonaceous particles and free and fused polycyclic aromatic hydrocarbons (PAHs) which are formed after the incomplete combustion of gasoline, diesel, and other petroleum fuels (Boffetta et al., 1997; Niranjan and Thakur, 2017). PAHs in soot are considered to be the main carcinogenic compound (Boffetta et al., 1997; Wang et al., 2001). Among many soots, diesel exhaust particles are considered as the main source of environmental soot and responsible for the increased rate of morbidity and mortality in humans (Reynolds and Richards, 2001; Patel et al., 2013). The carbon particles from wood smoke and road traffic also produce allergic adjuvant effects and exacerbate lung-associated disorders (Samuelsen et al., 2008). Other model soots, such as fullerene soot (carbon black and nanocaged structure), also share the same kind of toxicological responses with environmental soot. However, the role of both types of soots in causing biological effects on the respiratory system is not extensively investigated (Baierl et al., 1996; Harhaji et al., 2007). Nevertheless, some mechanisms related to biological effects are known for them. For example, environmental soot (like diesel exhaust particles, DEP) significantly induces reactive oxygen species (ROS) in the lungs of humans along with experimental animals (Hussain et al., 2009, 2010). Diesel exhaust particulate matter also triggered the apoptosis of lung airway cells in a zinc transporter-dependent manner (Ackland et al., 2007). An alteration of monocyte differentiation was also observed in *in vitro* cultured monocytes in response to DEP (Chaudhuri et al., 2012). TRPA1 is activated by diesel exhaust particles, giving important information on the relationship of chemical composition and toxicity. On the other hand, ROS generation can lead to autophagy of cells (Altman and Rathmell, 2009; Zhou et al., 2011). It is also widely accepted that nanoparticles induce autophagy in a variety of models by more than one mechanism (Afeseh Ngwa et al., 2011). Notably, soot also exists in nanoparticle form and therefore may affect autophagic mechanisms in various ways (Andon and Fadeel, 2013). Considerable studies have described that a defect in autophagy is connected to a development of lung pathologies; however, the underlying mechanisms are still unknown (Aggarwal et al., 2016). Thus, to understand the biological effect and fundamental mechanism of soot on the lungs, the present study was designed, where fullerene soot and diesel soot were used first on lung epithelial cells to know the biological effect on cell viability, and later the observations were assessed in an *in vivo* system to investigate the effect of soot on lung tissue. We found that in the presence of fullerene soot, lung epithelial cells show proliferation by reducing autophagy. This effect was found to be dependent on the Akt pathway. The same effect was observed in mouse lung cells where tissue proliferation and underexpression of autophagic genes were evident. Thus, we have shown that soot-induced cell proliferation can cause a hyperplasia-like tissue development, which may in turn lead to tissue remodeling and lung diseases. Our work thus projects a glimpse into the consequences of soot exposure to human lungs.

## Materials and Methods

### Chemicals and Reagents

Fullerene soot was obtained from Sigma-Aldrich (product number 572497, St. Louis, MO, United States). Primary rabbit polyclonal antibodies against cleaved caspase-3 and light chain 3 (LC3) were procured from Cell Signaling Technology (Danvers, MA, United States). APG-5, Ki-67, Beclin-1, p53, and secondary anti-rabbit HRP-conjugated antibodies were procured from Santa Cruz Biotechnology (Santa Cruz, CA, United States). Primary rabbit polyclonal anti-p62 antibodies and secondary anti-rabbit Alexa Fluor^®^ 488-conjugated antibodies were purchased from Life Technologies (Invitrogen, Carlsbad, CA, United States). All other chemicals were purchased from Sigma-Aldrich (St. Louis, MO, United States). Diesel exhaust particles were collected using the standard procedure as described before with desired modifications (Gangwar et al., 2011).

### Cell Lines Used

Human lung epithelial cell line, A549, and human embryonic kidney cell line, HEK 293, were used in the study. All these cell lines were purchased from the National Centre for Cell Sciences, Pune, India, and since then continuously being maintained in our laboratory.

### Culture and Passaging of the Cells

Dulbecco’s Modified Eagle’s Medium or DMEM-Ham’s F12 nutrient mixture was used for culturing the cells. In this nutrient mixture medium, 10% heat-inactivated fetal bovine serum was added. The culture of the cells was maintained at 37°C in a humidified atmosphere of 5% CO_2_/95% air. The pH of the medium was adjusted to nearly 7.4 with the help of 0.1 N NaOH/0.1 N HCl followed by filter sterilization by Merck Millipore filters of pore size 0.22 μm. At first, sterility of the medium was checked, keeping the medium in a CO_2_ incubator for at least 48 h. Subculture of the cells was done using trypsinization as per the requirements. Depending on the confluence, cell suspension was distributed into two to three flasks and 4 ml fresh medium was added to each flask.

### Preparation of Suspension of Fullerene Soot and DEP in Medium

Fullerene soot was obtained from Sigma; a stock of the fullerene soot (4 mg in 2 ml) was directly made in the medium (DMEM: Ham’s F12). In a similar way, DEP was also dissolved directly in the medium. The further dilutions of the required concentrations (125 to 2,000 μg/ml) were made in the medium (Lankoff et al., 2017). This range of concentrations was selected as per the availably in the existing literature (Al-Sadik et al., 2020). All these concentrations are non-toxic concentrations, as they did not cause any cell death (Nielsen et al., 2008). A dose-dependent study was conducted for the assessment of non-cytotoxic concentrations. The dissolution of the soot was enhanced by vortexing it many times with regular intervals for a period of 1 h after mixing. Exposure to the cells with different concentrations of soots was done inside a biosafety cabinet for various time periods as per the requirements of the experiments.

### Cell Viability Assay by MTT

MTT assay was performed to determine cell viability (Mosmann, 1983). Cells (4 to 8,000/well) were seeded in a 96-well plate. After exposure with different concentrations of soots or inhibitors (as mentioned in individual section “Results”), MTT salt 3-(4,5-dimethylthiazol-2-yl)-2,5-diphenyltetrazolium bromide (20 μl/well containing 100 μl of cell suspension; 5 mg/ml in PBS) was added. Color was read at 530 nm, using a multi-well microplate reader. For each group, a minimum of three to six replicates were used.

### Cell Proliferation Assay Using a Fluorescence-Based Kit

Cell proliferation in culture was measured by the CyQUANT^®^ cell proliferation assay kit as per the protocol described by the provider (Thermo Fisher Scientific, Waltham, MA, United States). In brief, 4,000 cells per well were seeded in the 96-well plate and were treated with different soot concentrations (ranging from 50 to 2,000 μg/ml). Cell proliferation was determined by using the kit procedure as per the manufacturer’s instructions.

### Measurement of ROS Generation

Measurement of intracellular generation of ROS was accomplished using fluorescence dye dichlorodihydrofluorescein diacetate (DCF-DA). A549 cells were seeded at the cell density of 1 × 10^4^ cells/well in 96-well plates. Following the treatment with soot (or inducer LPS 50 μg/ml and soot 250 μg/ml), the medium was aspirated and DCF-DA (10 μM final concentrations) in phenol red-free Hank’s balanced salt solution (HBSS) buffer (100 μl/well) was added to the plate. A minimum incubation period of 30 min was given in the dark at 37°C in a CO_2_ incubator. Fluorescence measurement was done at excitation of 485 nm and emission of 530 nm, by a fluorescence reader (Perkin Elmer, Waltham, MA, United States).

### Measurement of Soot Internalization Using Methanol Treatment

The determination of internalization of soot was accomplished by methanol treatment of soot. Initially, soot was weighed and mixed with methanol, and then it was vortexed for 1 h intermittently for mixing. Fullerene soot was incubated with methanol for 24 h, and then it was coated in the 96-well plates having coverslips in their bottom. These plates were allowed to dry in the laminar air flow for a period until the extra methanol is evaporated and the plates have only fullerene soot. A549 cells were then plated on the coverslips and allowed for a period of 24 h for the attachment. After the incubation period, the cells were fixed with paraformaldehyde at 4°C. Internalization of fullerene soot was measured using a fluorescence microscope. Internalized particles give red fluorescence, which were captured using a fluorescence microscope.

### Measurements of Organic Carbon and Elemental Carbon by Using the OCEC Instrument

The measurement of organic carbon and inorganic carbon contents before and after the soot treatment was done using the semi-continuous OCEC carbon aerosol analyzer as per the manufacturer’s protocol (Sunset Laboratory Inc., Tigard, OR, United States). In brief, A549 cells (1 × 10^5^/ml) were exposed to fullerene soot (1,000 μg/ml) for different time intervals (4, 8, 12 h). Cells were then collected and lysed in lysis buffer containing 150 mM NaCl. The lysed cells were then centrifuged at 400g for removal of debris. Cell debris was discarded, and supernatants were kept at the filters for analysis using the OCEC instrument. The experiment was conducted with duplicates and repeated once.

### Animal Studies

Eight- to twelve-week-old BALB/c mice were used in this study. Initially, mice were divided in two groups, namely, control and soot (fullerene soot)-treated mice. Each group consisted of at least four mice. Institutional animal ethical clearance was obtained to conduct the use of experimentation on the mice as per the Committee for the Purpose of Control and Supervision of Experiments on Animals (CPCSEA) guidelines. Mice were anesthetized using isoflurane and then intranasally challenged with fullerene soot. One hundred micrograms of soot prepared in saline (by mixing with saline using a vortex) was given to mice per dose in 50 μl of volume. Two intranasal doses were given to the mice in a 12-day period, in an interval of 6 days. At last, mice were sacrificed and organs were isolated. Tissues were fixed in neutral buffered formalin and later were analyzed for different markers.

### Immunocytochemistry for Protein Localization Studies

Six-well plates containing coverslips at their bottom were used to seed the cells (2 × 10^5^/well). Treatment of either soots or inhibitors was given after 24 h. After the incubation period, cells were fixed with 4% paraformaldehyde. Subsequently, cells were treated with 0.05% H_2_O_2_ in methanol for 1 h at room temperature in the dark with mild agitation to make them permeable. Cells were blocked by blocking buffer (0.02% BSA + 0.002 % Triton X-100) for a period of 30 min at room temperature. After blocking, treatment with primary antibodies at a dilution of 1:100 was given to the cells with mild agitation overnight at 4°C. Cells were then treated with secondary Alexa Flour 488-conjugated antibodies, at 1:200 dilutions in blocking buffer for 1 h at room temperature with mild agitation. The cells were washed with PBS after each step. Images were captured by upright fluorescence microscopy at different magnifications.

### Immunohistochemistry of the Tissue Sections

The tissues of mice were fixed in neutral buffered formalin, embedded in paraffin, and cut into 5-μM-thin sections. Sections were fixed to positively charged slides. The endogenous peroxidase activity in the tissues was quenched using 0.3% hydrogen peroxide in methanol. Sections were blocked by non-specific protein blocking with 1% BSA. Tissue sections were then incubated with different antibodies overnight at 4°C at 1:100 dilutions, followed by 1:200 dilutions of HRP-conjugated anti-rabbit IgG secondary antibody for 1 h at room temperature. Subsequently, these slides were developed using DAB Kit and further counterstained with Nuclear Fast Red or hematoxylin. Negative controls include a secondary antibody alone without the primary antibody.

### Western Immunoblotting Analysis for the Expression of Proteins

Cell lysates of A549 cells after incubation with different treatments (controls and exposed) were prepared in 300 μl of lysis buffer, containing 50 mM Tris–HCl, 1 mM EDTA, 100 mM NaCl, 100 μg/ml phenylmethylsulfonyl fluoride (PMSF), and protein inhibitor cocktail. The obtained lysates were then centrifuged at 10,000 g for 5 min at 4°C. Protein estimation in supernatant was estimated using the BCA Kit provided by Pierce (Thermo Fisher Scientific). The linear range values of protein estimation were used to calculate the exact concentrations using different dilutions. Samples were mixed with NuPAGE and 4× loading buffer containing 200 mM dithiothreitol (DTT). In most of the cases, 25 to 100 μg of protein was separated on 8–12% SDS-PAGE and subsequently transferred to the PVDF membranes for the particular analysis. The blocking of membrane was done by blocking buffer (5% non-fat dry milk, 100 mM NaCl, 10 mM Tris pH = 7.5, 0.1% Tween 20) for 1 h at room temperature. Standardization of the signal was done using different concentrations of protein or antibodies to avoid saturation effects and to get optimized results in a linear range. Membranes were then treated with different protein-specific primary antibodies (at 1:500 to 5,000 dilutions) at 4°C overnight. Membranes were washed five times with TBST. Membranes were then treated with HRP-conjugated secondary antibodies for 1 h (from 1: 2,000 to 1: 10,000 dilutions) at room temperature. After that, blots were developed by using the ECL (enhanced chemiluminescence) system provided by Pierce. Densitometry analyses of bands were accomplished by the gel documentation system (Bio-Rad, Hercules, CA, United States).

### Statistical Analysis

Results from majority of the experiments are expressed as mean ± SEM or otherwise specified. The accomplishment of statistical analysis was done by one-way analysis of variance (ANOVA), followed by a Newman–Keuls *post-hoc* test or otherwise specified. The *p* value < 0.05 was considered statistically significant. GraphPad Prism 5 software was adopted for the statistical analysis.

## Results

### Effect of Fullerene Soot on the Cell Morphology, Cell Proliferation, and Apoptosis of Lung Epithelial Cells, A549

**FIGURE 1:**
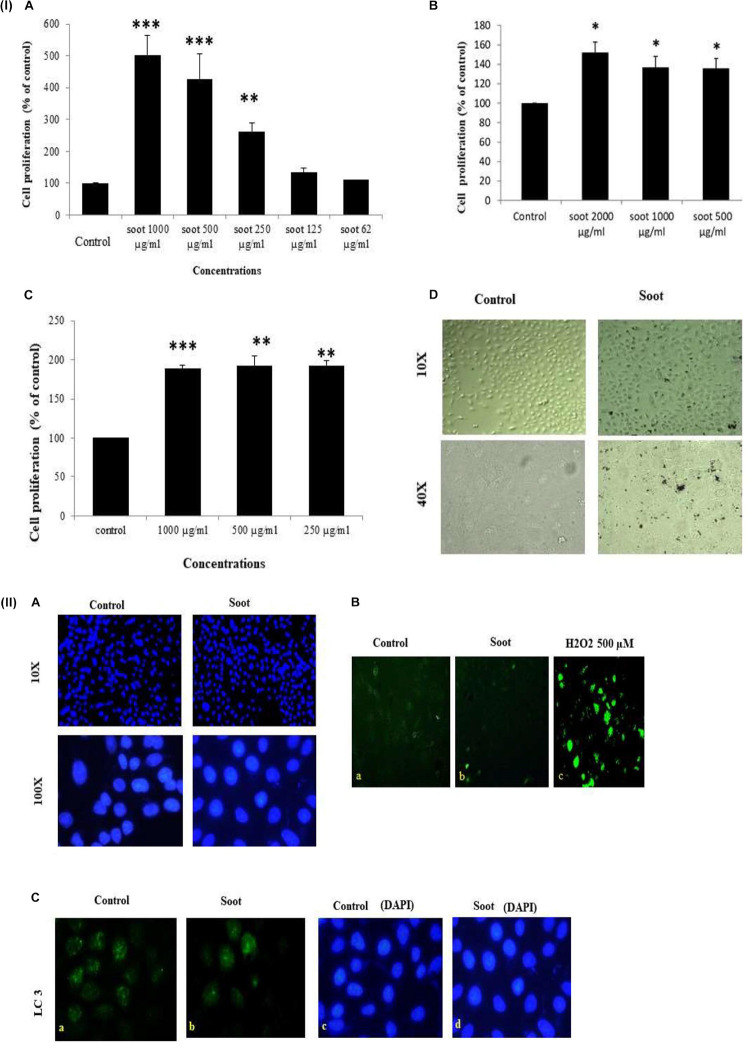
(I) Effect of soot on cell proliferation of A549 cells. Histograms represent mean ± SEM of different treatment groups. **(A)** Dose response of fullerene soot on A549 cells in regular medium (DMEM:Ham’s F12+ 10% FBS) measured by MTT. **(B)** Dose response of fullerene soot (dispersed in the phosphate buffer saline) on proliferation of A549 cells was measured by MTT assay. **(C)** Dose-dependent study of fullerene soot on A549 cells in glucose-free (GF) medium measured by a kit (fluorescence method). **(D)** Bright-field images of soot exposed A549 human lung epithelial cells. The top panel shows the lung epithelial A549 cells at ×10 original magnification while the lower panel shows the control and soot-exposed cells at ×40 original magnification. ^∗^*p* < 0.05 treated vs. control, ^∗∗^*p* < 0.01 treated vs. control, ^∗∗∗^*p* < 0.01 treated vs. control. Data shown here are one representative experiment out of three. (II) Effect of soot on cell death of A549 cells. **(A)** DAPI-stained control and soot exposed cells. **(B)** Cleaved caspase-3 expression in A549 cells in response to soot. Immunofluorescence photomicrographs of A549 cells are shown after 24 h of H_2_O_2_ exposure of cells. Green fluorescence shows the cleaved caspase-3 expression in A549 cells (at ×40 original magnification). H_2_O_2_ 500 μM was used as a positive control for measurement of cleaved caspase-3. **(C)** LC3 punctate staining, in A549, cells. The right panel shows DAPI staining for the nucleus. The left panel shows LC3 immunostaining.

At first, we tested the effect of fullerene soot on lung airway epithelial cells, the first site of entry of soots to the human body. In order to assess the toxic effect of fullerene soot, we tested its effect on the cell viability or cell proliferation of A549 cells. As seen in Figure 1 (IA), soot exposure did not cause the death of these cells. Soot concentrations (250, 500, and 1,000 μg/ml) have increased the MTT score in 24 h of incubation period of the cells. As shown in Figure 1 (ID), soot did not cause the death of these cells. Morphologically, soot-exposed cells are big in size and different from control cells. Notably, some particles of soot are seen to be internalized by the cells exposed to fullerene soot. In addition to MTT assay, we also tested fullerene soot-induced apoptosis of A549 cells. Apoptosis of A549 cells in response to soot was measured by DAPI nuclear staining. To achieve this, 1,000 μg/ml (middle of the concentrations showing cell proliferation) of concentration was exposed to the lung A549 cells for a period of 24 h. As seen in Figure 1 (IIA), soot did not induce apoptosis, as soot exposure to cells did not cause any DNA fragmentation inside the cell nucleus. Mechanistically, at the molecular level to assess soot-induced protection from apoptotic cell death, we measured cleaved caspase-3 expression (a marker of apoptosis). Figure 1 (IIB) shows that soot exposure did not induce the cleaved caspase-3 level in A549 cells in 24 h of the exposure period and minimal expression was comparable to control. These data further confirmed, in previous results of DAPI staining Figure 1 (IIA), that soot does not induce apoptosis of A549 cells and may be involved in the cell proliferation. Subsequently, based on our speculation that soot may work as a carbon source to these cells or induce protective mechanisms, we have further tested the effect of soot on the cell proliferation of A549 cells in glucose-deprived (glucose-free medium) conditions. Different concentrations (500, 1,000, and 2,000 μg/ml) of soot were exposed to lung A549 cells for a period of 24 h. As seen in Figure 1 (IC), soot significantly induced cell proliferation of A549 cells as compared to control cells. Additionally, to assess autophagic cell death, we measured light chain-3 (LC3) expression by seeing the appearance of punctate staining in A549 cells in response to soot. As seen in Figure 1 (IIC), soot exposure to cells did not cause LC3 expression in cells and thus it is confirmed that soot did not cause any autophagic cell death in the A549 lung epithelial cells. Fullerene soot (2,000 μg/ml) also induced cell proliferation in phosphate-buffered saline alone (Supplementary Figure 1).

### Fullerene Soot Inhibits Autophagic and Apoptotic Cell Death of Human Lung Epithelial, A549 Cells

**FIGURE 2:**
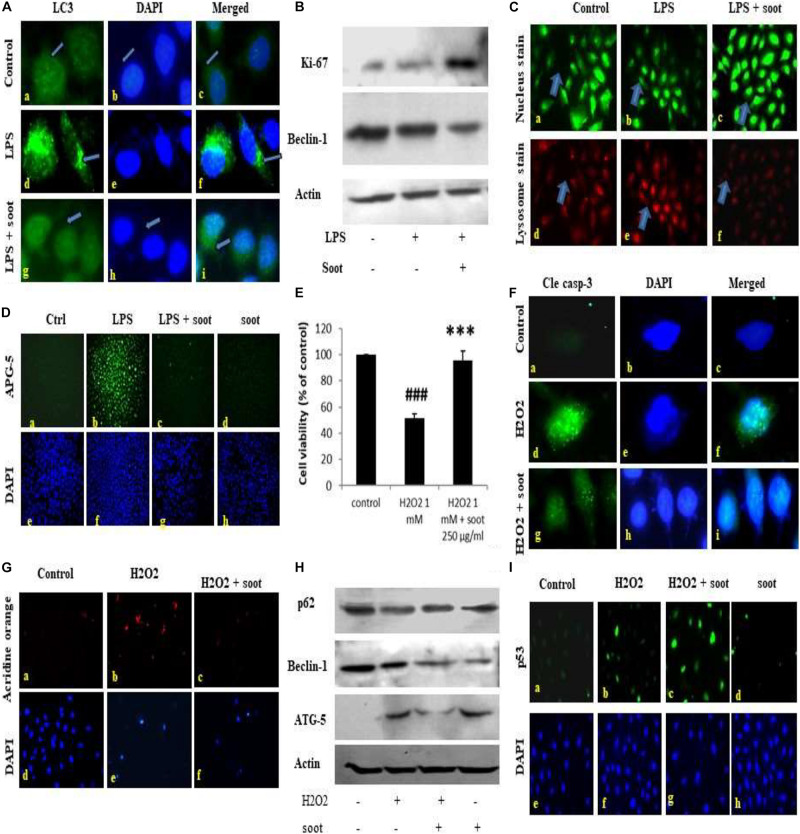
Fullerene soot inhibits LPS-induced autophagy and H_2_O_2_-induced cell death of A549, cells. **(A)** Effect of LPS on LC3 punctate staining in A549 cells in the 24-h exposure. **(A–C)** Immunofluorescence photomicrographs of control (no LPS) A549 cells are shown. Green fluorescence shows LC3 punctate staining (×100). **(D–F)** LC3 punctate staining in LPS (10 μg/ml) treated cells. (G–I) LC3 punctate staining in LPS + fullerene soot-treated cells. **(B)** Western immunoblotting profile of soot on LPS-induced human lung epithelial cells. **(C)** Effect of soot on LPS induced acridine orange staining in A549 cells (at ×40). **(D)** Effect of fullerene soot on LPS-induced ATG-5 expression profile in human lung epithelial cells (at ×10). **(E)** Effect of fullerene soot on the H_2_O_2_-induced cell death of A549 cells as measured by the MTT assay. **(F)** H_2_O_2_-induced cleaved caspase-3 expression is inhibited by the fullerene soot (×100). The left panel shows green fluorescence showing expression of cleaved caspase-3, the middle row shows the counterstaining by DAPI, and the right panel shows the merged images. **(G)** Acridine orange staining in response to H_2_O_2_-induced autophagic response (×20). **(H)** Western immunoblotting profile of the proteins associated with the autophagic and apoptosis genes. **(I)** Expression profile of p53 in response to H_2_O_2_ and soot. The upper panel shows the p53 immunostaining, and the lower panel shows the nuclear staining by the DAPI (×20). ^###^*p* < 0.001 compared with control. ***p < 0.001 compared with H_2_O_2_ treated group.

Fullerene soot did not decrease cell viability (or cause apoptotic or autophagic cell death) and caused cell proliferation of A549 cells. This suggested us that soot may be inhibiting autophagy and thus inducing cell proliferation. To test this hypothesis that “soot inhibits autophagy,” we used LPS (lipopolysaccharide) to induce autophagy in A549 cells and measured the inhibition of LPS-induced autophagy due to soot. To accomplish this, A549 cells were exposed to LPS alone and in combination with fullerene soot. Measurement of autophagy was done by observing LC3 punctate staining. As seen in Figure 2A, LPS profoundly induced autophagy in A549 cells by showing LC3 punctate staining. Fullerene soot inhibited LPS-induced punctate staining in A549 cells (Supplementary Figure 2). The protein expression profile of autophagic genes was also measured in response to LPS alone or in combination with soot. Fullerene soot changed the expression profile of LPS-induced autophagic genes, i.e., beclin-1 and ATG-5. Apart from autophagic genes, we have also tested the proliferation-responsive genes in the presence and absence of soot and LPS. As seen in Figure 2B, soot upregulated the protein expression profile of the Ki-67 (a marker of cell proliferation) gene and thus shows that soot induced proliferation (see Supplementary Figure 3 for quantitative expression). Soot has also inhibited LPS-induced lysosome number (as measured by acridine orange dye) very profoundly (Figure 2C).

In addition to autophagic cell death, we also measured the inhibition of apoptosis by fullerene soot. To measure the inhibition of apoptotic cell death, we tested the inhibition of H_2_O_2_-induced cell death (reduction in the cell viability) by soot. To accomplish this, A549 cells were exposed to H_2_O_2_ (500 μM) alone and in combination with soot (1,000 μg/ml). Assessment of apoptosis was done by measurement of the expression profile of cleaved caspase-3, DAPI nuclear fragmentation staining, and measurement of apoptosis-associated proteins. As seen in Figure 2F, H_2_O_2_ significantly stimulated cleaved caspase-3 expression as measured by immunocytochemistry and also showed nuclear fragmentation as seen by DAPI staining. Soot inhibited cleaved caspase-3 and nuclear fragmentation. The Western immunoblotting profile shows that soot inhibited the expressions of autophagic and apoptosis proteins stimulated by H_2_O_2_ exposure. As seen in Figure 2H, soot changed the beclin-1 and ATG-5 protein expression. showing that H_2_O_2_-induced autophagy is inhibited by fullerene soot exposure. Interestingly, soot also inhibited the H_2_O_2_-induced lysosome number (Figure 2G) and potentiated p53 expression (Figure 2I).

This study confirmed that soot is a major inhibitor of autophagic and apoptotic cell death.

### Fullerene Soot Inhibits Starvation-Induced Autophagy and Induces Proliferation of A549 Cells, Which Is Reversed by Akt-Inhibitor X

**FIGURE 3:**
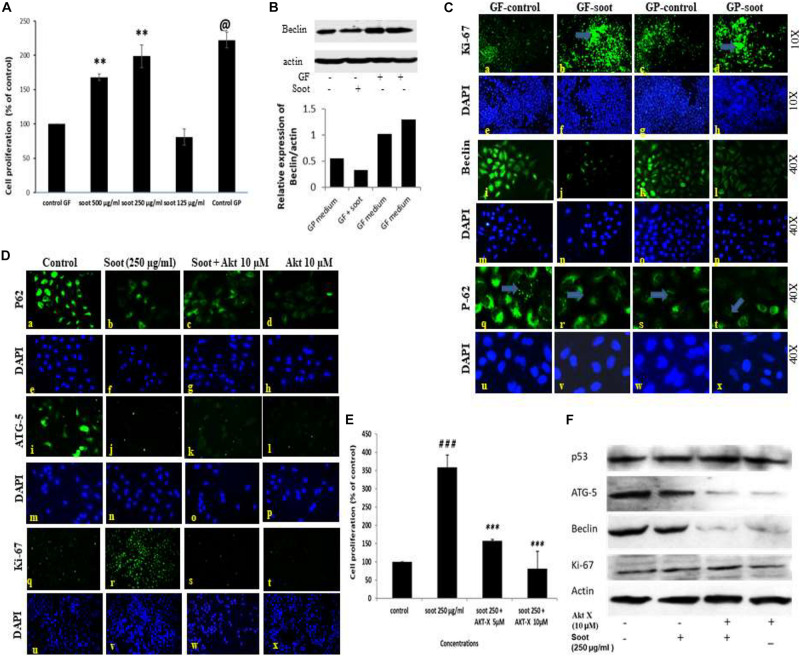
Fullerene-soot inhibited starvation-induced autophagy in A549 cells, which is reversed by Akt Inhibitor X. **(A)** Cell proliferation assay in the presence and absence of fullerene soot in glucose-free medium. ^∗∗^*p* < 0.01, @*p* < 0.001 compared with control GF. **(B)** Western immunoblotting of beclin due to exposure of soot in the presence and absence of glucose (top), histograms representing densitometric intensity of bands (down). **(C)** (a–d) expression profile of Ki-67 (green fluorescence) in the presence and absence of soot in the mediums with and without glucose. a, glucose-free (GF) medium without soot; b, glucose-free medium with soot; c, medium with glucose; d, medium with glucose with soot. (i–l) expression profile of Beclin-1. i, glucose-free medium; j, glucose-free medium with soot; k, medium with glucose; l, medium with glucose after soot exposure. (q–t) p-62 localization profile after the exposure of fullerene soot in glucose-free and glucose-containing medium. q, glucose-free medium; r, free medium with soot; S, medium with glucose; t, fullerene soot exposure in medium with-glucose. **(D)** Protein expression profiles of genes in glucose-free medium, after treatment with Akt Inhibitor X, by Immuno-cytochemistry. Expression profile of p62 (×20). a, glucose-free medium; b, medium with soot; c, soot exposure with Akt Inhibitor X (5 μM); d, soot with Akt Inhibitor X (10 μM). ATG-5 expression profiles (× 20), (i–l); i, glucose-free (GF) medium; j, medium with soot; k, soot exposure with Akt inhibitor (5 μM); l, soot exposure with Akt Inhibitor X (10 μM). Ki-67 expression profile (× 10). q, glucose-free medium; r, medium with soot; s, fullerene soot exposure with Akt Inhibitor X (5 μM); t, soot exposure with Akt Inhibitor X (10 μM). DAPI staining (blue image) is shown below, respectively, in each green fluorescence image. **(E)** Effect of Akt Inhibitor X on the cell proliferation of A549 cells due to soot in glucose-free medium. ^###^*p* < 0.01 compared with control and ^∗∗∗^*p* < 0.001 compared with the fullerene soot-treated group. **(F)** Western immunoblotting of autophagic proteins in response to Akt Inhibitor X.

It is well established that glucose starvation of cells induces autophagy in cells (Nakai et al., 2020). Therefore, we tested whether soot treatment can inhibit starvation-induced autophagy. To accomplish this, A549 cells were starved of glucose for 24 h and assessment of autophagy parameters was carried out in different conditions. Prior to starting the mechanistic study of starvation-induced inhibition of autophagy, a dose-dependent study in glucose-free medium was conducted (Figure 3A). As seen in Figure 3A, 250 μg/ml of soot induced more proliferation in glucose-free medium than 500 μg/ml of soot. We used 250 μg/ml of fullerene soot concentrations in subsequent experiments in glucose-free medium. Also the expression of beclin protein occurs in similar manner (Figure 3B). As seen in Figure 3C, starvation of cells has significantly upregulated autophagy in lung epithelial (A549) cells, as seen by the upregulated protein expression profile of autophagy genes beclin-1, p62, and LC-3. Soot exposure (has significantly inhibited the starvation-induced beclin-1 expression. Glucose starvation of the A549 cells has significantly upregulated p62 punctate staining in a 24-h duration time period. Apart from autophagy genes, proliferation-responsive gene Ki-67 was also upregulated due to soot exposure in a 24-h period. Soot significantly inhibited autophagy in A549 cells, as seen by the LC3 punctate staining.

The Akt signaling pathway is associated with the starvation-induced autophagic response, but the role of soot on Akt pathway activation was not known. To understand the mechanism of soot-associated cell proliferation and autophagic inhibition, we tested the role of Akt Inhibitor X on soot-induced cell proliferation and autophagic response. Cells were treated with different concentrations of soot in the absence of glucose, and the effect of Akt Inhibitor X was measured on the cell proliferation and autophagic response. Soot exposure significantly stimulated cell proliferation of human lung epithelial cells A549 in 24 h. As demonstrated in Figure 3D, it was seen that Akt Inhibitor X (5 and 10 μM) has significantly inhibited soot-induced cell proliferation of human lung epithelial cells in a dose-dependent manner. In addition to the inhibition of cell proliferation, the Akt inhibitor also showed downregulation of protein expressions of autophagy genes, i.e., p62, beclin-1, and ATG-5, in a dose-dependent manner Figure 3F. Soot exposure also inhibited p62 punctate staining in the cells showing inhibition of autophagic flux.

### The Diesel Soot and Biomass Burning Soot Also Induced Cell Proliferation of A549 Cells and Affected Autophagic Mechanisms, Which Are Not Reversed by Akt Inhibitor X but by Chloroquine

**FIGURE 4:**
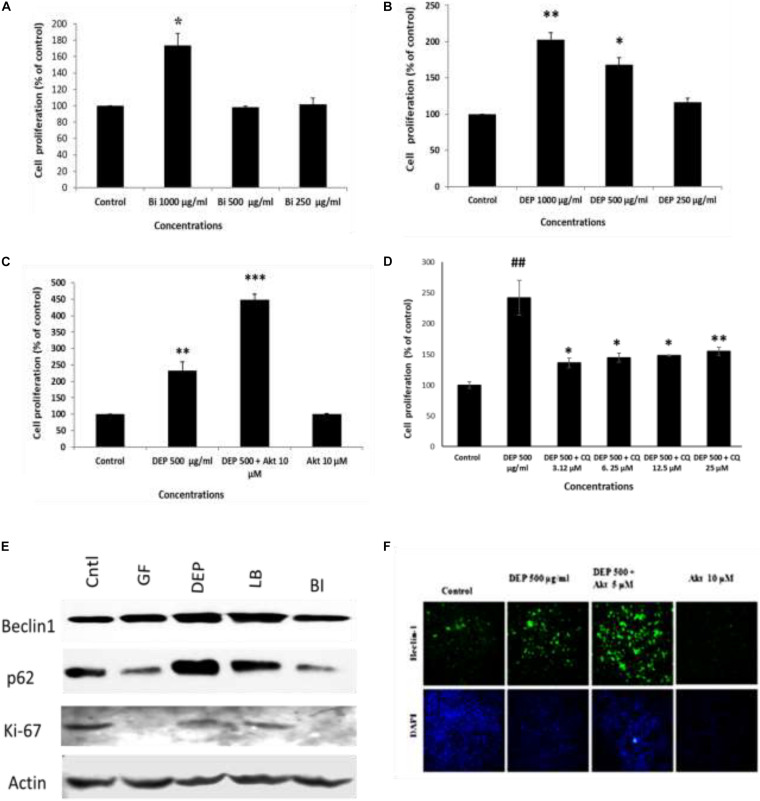
Dose-dependent study of DEP (diesel exhaust particles) and biomass burning soot on the cell proliferation of A549 cells; effect of autophagic inhibitor chloroquine and Akt Inhibitor X. Histograms represent the mean ± SEM of the different treatment groups. **(A)** Dose response of biomass burning soot on the A549 human lung epithelial cells in glucose-free medium measured by MTT assay. **(B)** Dose response of diesel exhaust particles, on A549 human lung epithelial cells in glucose-free medium measured by MTT assay. **(C)** Effect of Akt Inhibitor X, on the diesel exhaust particle-induced cell proliferation. **(D)** Effect of chloroquine (an inhibitor of autophagic flux) on the DEP-induced cell proliferation of A549 cells. **(E)** Western immunoblotting profile of different autophagic proteins in response to different kinds of soots, i.e., DEP, diesel exhaust particles; BI, soot from biomass burning (wood burned); LB, stands for soot from burned leaf; GF, glucose-free medium. **(F)** Effect of Akt Inhibitor X, on the diesel exhaust particle-induced beclin-1 expression profile. ^∗^*p* < 0.05, ^∗∗^*p* < 0.01, and ^∗∗∗^*p* < 0.001 compared with control in panels A–C. ^#^*p* < 0.05, ^#^*p* < 0.01 compared with control, and ^∗^*p* < 0.05, ^∗∗^*p* < 0.01, and ^∗∗∗^*p* < 0.001 compared with DEP- or soot-treated group in panels D, E.

Furthermore, to understand the effect of ambient soot, the effect of diesel soot (diesel exhaust particles or DEP) and biomass burnt (BI) soot (wood burning) was evaluated on A549 cells. As seen in Figure 4A, biomass burning soot did not cause cell death (reduction in cell viability) of lung airway epithelial A549 cells. Soot concentrations (250, 500, and 1,000 μg/ml) increased the MTT score at 24 h, showing cell proliferation of lung epithelial A549 cells. Similarly, we also tested the effect of diesel exhaust particles. Lung A549 cells were exposed to different concentrations (250, 500, and 1,000 μg/ml) of DEP for a period of 24 h. As seen in Figure 4B, DEP significantly caused the cell proliferation of A549 cells as compared with control cells. In addition to cell proliferation, we also tested the expression profiles of autophagic proteins in response to DEP and biomass-burnt soot. As shown in Figure 4E, soot exposure to A549 cells significantly upregulated the expressions of autophagic proteins in response to both types of soot. We have also tested the role of Akt Inhibitor X on DEP- and BI-induced cell proliferation and autophagic responses. Cells were treated with different concentrations of DEP and BI and in the absence of glucose, and the effect of Akt Inhibitor X was measured on the cell proliferation and autophagic responses. DEP and BI soot exposure to the A549 cells significantly stimulated cell proliferation in 24 h. Interestingly, as demonstrated in Figure 4C, Akt Inhibitor X (10 μM) did not inhibit the DEP-induced cell proliferation of human lung epithelial cells. In fact, Akt inhibitor treatment significantly potentiated the DEP-induced cell proliferation. Co-exposure of Akt Inhibitor X with DEP also showed an increase in the expression of autophagy protein beclin-1 in a dose-dependent manner. However, chloroquine, an inhibitor of autophagic flux, has significantly inhibited DEP-induced cell proliferation of A549 cells (Figure 4). Similarly, autophagic inhibitor 3-MA has also significantly inhibited DEP-induced cell proliferation in a dose-dependent manner (Supplementary Figure 4).

### Fullerene Soot Particles Are Internalized and Co-localizes With Autophagosome-Associated Protein p62

**FIGURE 5:**
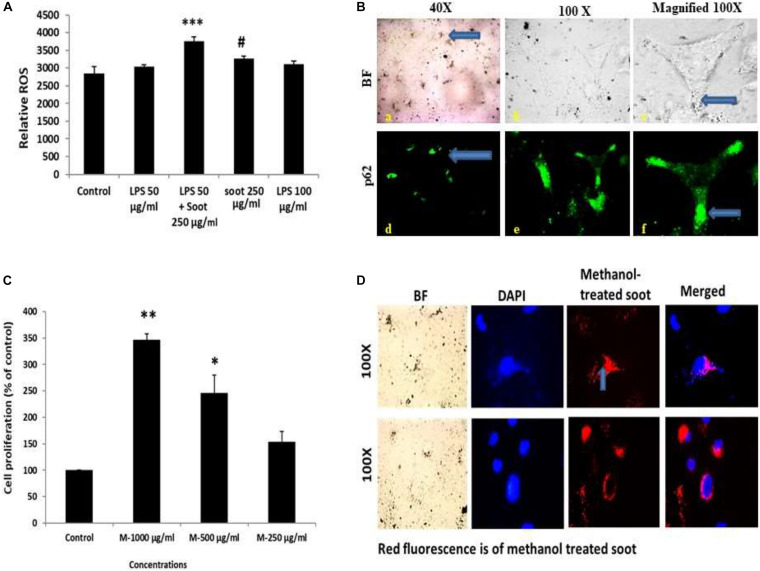
Fullerene soot enters the cell and co-localize with the autophagic protein p62. **(A)** Histograms represent comparative ROS formation in the A549 cells. ^∗∗∗^*p* < 0.001 compared with control and #*p* < 0.05 compared with LPS + fullerene-treated group. **(B)** Cellular localization of soot with the autophagy protein p62. Green fluorescence images show the p62 localization. Photomicrographs were captured at ×40 and ×100 original magnifications. The upper panel corresponds to the bright-field images, and the lower panel shows the p62 localization as a green fluorescence. **(C)** Dose-dependent study of methanol-treated fullerene soot on the cell viability of A549 cells. ^∗^*p* < 0.05, ^∗∗^*p* < 0.01 compared with control. **(D)** Measurements of the internalization pattern of soot inside the cell. Red fluorescence shows methanol-treated soot inside the cell. The corresponding DAPI photographs are also shown with the photograph to position the cell nucleolus.

We speculated that soot-associated autophagic response is mediated by its physical presence with the autophagosomes. To test this speculation, we have first coated six-well plates with methanol-treated fullerene soot and allowed the cell to grow on the coated plates. After the incubation period of 24 h, the cells were fixed and seen under a fluorescence microscope. As seen in Figure 5D, cells have internalized some particles of fullerene soot (reflected in the form of red fluorescence). To measure the interaction of these fullerene soot particles, we also tested the p62 expression and localization pattern in soot-treated cells. Figure 5B shows that p62 co-localizes with the internalized soot particles. Additionally, to confirm that treatment with methanol did not alter the cell proliferation-associated effect of the lung epithelial cells, we have also tested the effect of methanol-treated soot on the cell proliferation of A549 cells. The methanol-treated soot concentrations 250, 500, and 1,000 μg/ml increased the MTT score at 24 h of incubation period. The data in Figure 5 denote that soot in fact caused the cell proliferation of lung epithelial A549 cells. It was interesting to note that soot particles co-localize with the autophagy proteins, suggesting its role in autophagy.

### Soot Induces Cell Proliferation and Inhibits Autophagy in the *in vivo* Model of Mice

**FIGURE 6:**
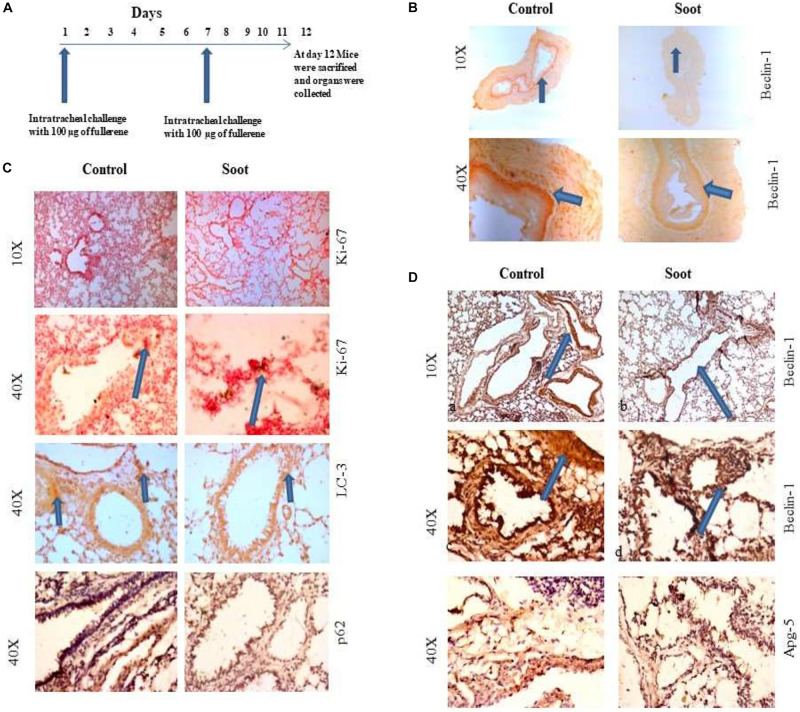
Assessment of soot-induced autophagic response in the mice. **(A)** The schematic representation of challenge to the mice with fullerene soot. Vertical arrows show the day of exposure to the mice. The horizontal line shows number of days of progression of experiment. **(B)** Beclin-1 expression in the esophagus of mice. a, esophagus section of saline treated mice; b, esophagus section of fullerene soot-treated mice. **(C)** Ki-67 and LC3 expression pattern in the lung section of saline-treated and soot-treated mice. **(D)** Beclin-1 expression in the lung section of mice. a, section of saline-treated mice; b, esophagus section of fullerene soot-treated mice; c and d are the same at ×40 magnification. ATG-5 gene expression profile in the lung and in the esophagus of saline- and soot-treated mice.

To further clarify that soot inhibited autophagy in *in vivo* conditions, we tested the effect of fullerene soot on autophagic response when it is intranasally delivered in mice. To accomplish this, 100 μg of soot was intranasally delivered to mice. A total of two intranasal challenges were given to mice in 12 days with an interval of 6 days (exposure plan of mice is depicted in Figure 6A. Control mice were delivered with saline alone. As seen in Figure 6C, basal autophagic markers were inhibited by the soot challenge. As seen in Figure 6C, fullerene soot has significantly upregulated Ki-67 protein expression in the lung airway epithelium, giving confirmation of the proliferation-inducing property of fullerene soot. Fullerene soot also downregulated autophagy-responsive protein beclin-1 in the lungs and in the esophagus, depicting its important autophagy-inhibiting properties. In addition to beclin-1 gene expression, fullerene soot has also downregulated the expressions of autophagy proteins LC3, p62, and APG-5 in the lung (Figure 6D). These results suggest that fullerene soot inhibits autophagy and induces cell proliferation also in *in vivo* conditions in healthy mice.

### Effect of Fullerene Nanoparticles on the Expression Profile of Autophagy Genes in HEK 293 Cells, p53 Translocation, and Organic Carbon Contents

**FIGURE 7:**
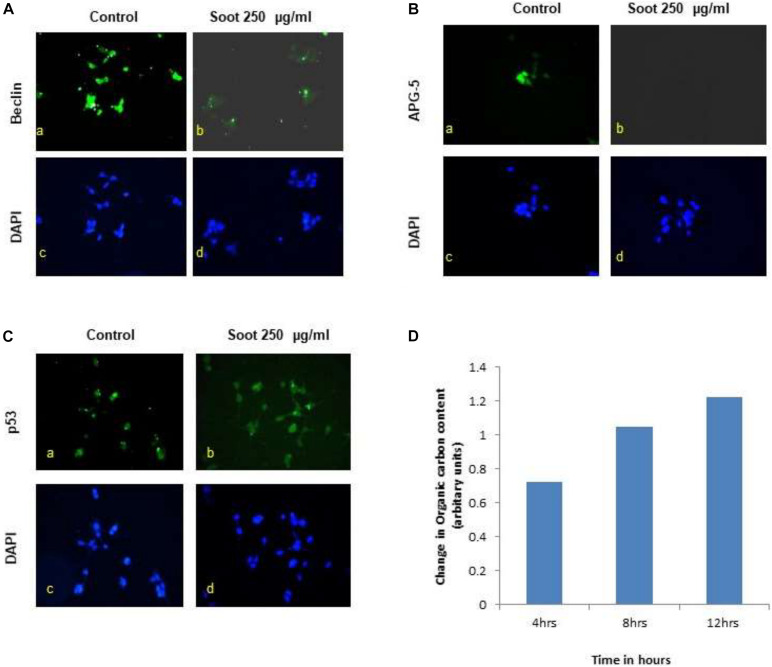
Effect of fullerene nanoparticles on the expression profile of autophagy (in HEK-293), organic carbon content, and p53 translocation. **(A,B)** Effect of fullerene soot in HEK-293 cells on beclin and ATG-5 expressions (in glucose free medium). **(C)** Effect of fullerene soot on translocation of p53 protein in HEK 293 cells. **(D)** Organic carbon content at the elemental level using the OCEC instrument was analyzed.

A549 is one of the immortalized cancerous cell lines and does not represent the characteristics of a normal cell type. Therefore, to understand the effect of fullerene soot, HEK 293 (non-cancerous cell line) was used as a non-cancerous cell line. Furthermore, the effect of fullerene soot on expressions of autophagic proteins in HEK 293 cells (Figure 7) was also assessed. As seen in Figures 7A,B, fullerene soot successfully attenuated the expressions of autophagic protein beclin and ATG-5 expressions (in glucose-free medium). It also illustrates that fullerene soot exposure did not cause any translocation of p53 protein in HEK 293 cells, depicting its non-carcinogenic effect (Figure 7C). As described in the previous results, fullerene soot induces cell proliferation and suppresses autophagy; it was desired to know whether fullerene soot particles are being used as a carbon source directly or indirectly by cells. To test this, organic carbon content (OC) at the elemental carbon (EC) level was measured using the OCEC instrument. As shown in Figure 7D, organic carbon content of fullerene soot-treated cells (lung epithelial A549 cells) increased with time as measured at different time intervals Figure 7D. This may indicate that elemental carbon of fullerene soot particles was utilized by cells as a carbon source. This may explain the mechanism of survival and cell proliferation of different cell types due to fullerene soot.

## Discussion

Exposure of environmental soot was thought to cause severe lung pathologies; however, their underlying mechanisms were not properly understood (Niranjan and Thakur, 2017). In addition to lung pathologies, the systemic effects of environmental soot are much more serious and affect many organs of the body including kidney, scrotal sac, brain, and cardiovascular functions (Chambellan et al., 2000; Niranjan and Thakur, 2017). In the present study, we demonstrate the effects of fullerene soot and DEP (diesel exhaust particles) on the proliferation of human lung epithelial and other cells. We show that both kinds of soot induce proliferation of lung epithelial cells *via* interruption of distinct autophagic mechanisms (Pidsley et al., 2018). Interestingly, soots did not induce cell death (reduction in MTT score or cell viability) of lung epithelial A549 cells. In fact, soots induced proliferation (increased MTT score, a measure of cell number) of lung epithelial A549 cells not only in *in vitro* culture conditions but also in mice (Shin et al., 2013). Our work in dissecting out distinct mechanisms of autophagy to regulate cell proliferation in response to exposure of soots provides an important insight in the pathological mechanisms of many soot-induced disorders.

It is important to know that autophagy is involved in the regulation of cell proliferation of many cell types. Autophagy regulator beclin-1 critically controls cell proliferation during tumorigenesis (Cicchini et al., 2014). Apatinib, a tyrosine inhibitor, decreases cell proliferation and initiates autophagy *via* the PI3K/Akt/mTOR pathway, establishing a link in the cell proliferation and autophagy regulation (Meng et al., 2020). Similarly, autophagy-dependent proliferation was seen in response to microRNA-107 in breast cancer cells (Ai et al., 2018). The present findings also align with these existing studies (Figures 1, 6) with multiple evidences at both cellular and molecular levels. In this study, autophagy is inhibited by fullerene soot, which may be a mechanism behind the induction of proliferation of lung epithelial cells, leading to lung dysfunctions (Qiang et al., 2014; Weng et al., 2014). Autophagy and cell proliferation are also linked with the morphological changes and apoptosis of cells, which we also found in response to soots (Chen et al., 2020). At the molecular level, soot did not induce cleaved caspase-3 expression Figure 1 (II), confirming its inability to induce type-I cell death (apoptosis) of the epithelial cells (Atale et al., 2014; Han et al., 2014). We also observed that soot did not cause DNA fragmentation in human lung epithelial cells and thus did not induce cell death (Gai et al., 2014).

One of the important conclusions that emanate from the current work is the fact that both soots do not cause cell death, which is evidenced by many lines of experiments (Figures 1, 2). In fact, it indicated their protective effects against cell death inducers. LPS is an endotoxin and is known to induce autophagy in different cells including lung epithelial cells by toll-like receptors (Waltz et al., 2011; Pei et al., 2015). Recently, it was shown that inhibition of LPS-induced autophagic response is linked with the cell proliferation in macrophage (Jakhar et al., 2014). Similarly, in the present study, LPS has significantly upregulated LC3 punctate staining in the cells, showing induction of autophagy (Figure 2). Treatment with fullerene soot clearly inhibited LPS-induced autophagic response and enhanced cell proliferation, showing the role of autophagy in the cell proliferation (reduction in lysosome number, Figure 2C). It is widely known that fullerene soot possesses antioxidant properties and may produce antioxidant-mediated affects (Dugan et al., 1996; da Silva Acosta et al., 2012). However, in the present study (as seen in Figure 5A), it was surprising to see that soot-mediated affects are not due to its antioxidant properties but preferably *via* autophagic mechanisms (Chan et al., 2006). In addition to this, fullerene soot has significantly upregulated the Ki-67 expression profile, confirming its proliferative potential at the molecular level (Troncoso et al., 2012). Hydrogen peroxide is known to cause apoptosis in many cells. H_2_O_2_ exerts its effect mainly by producing oxidative stress response. The oxidative stress may or may not be associated with the autophagic response of cells (Chen et al., 2008; Zhang et al., 2015). In the present study, H_2_O_2_ has significantly induced reduction in cell viability (cell death) by way of autophagy and apoptotic mechanism in human lung epithelial cells. Fullerene soot significantly inhibited H_2_O_2_-induced apoptosis and autophagic death. As shown in Figure 2D, fullerene soot also reversed H_2_O_2_-induced autophagy proteins beclin-1, p62, and ATG-5, showing its important mechanism of action but not by its antioxidant property. As seen in the section “Results” (Figure 5A), soot does not show its antioxidant properties at the used concentrations. It is clearly seen that H_2_O_2_ completed the autophagy and initiated the apoptotic events; however, soot alone did not induce autophagy. It is already known that H_2_O_2_ induces apoptosis *via* cleaved caspase-3 induction (Back et al., 2015; Li et al., 2015). In this study, soot inhibited H_2_O_2_-induced apoptosis and also inhibited cleaved caspase-3 expression and nuclear localization, suggesting its role at the transcription factor level. Soot also did not inhibit p53 expression, suggesting its less carcinogenic effects (Figure 2I).

Autophagic cell death is an important mechanism in nanoparticle toxicity (Chen et al., 2014; Loos et al., 2014). NLRP3 inflammasome and autophagy activation by metal nanoparticles was found to suppress cell proliferation (Sasabe et al., 2020). These metal nanoparticles (gold, silver, and palladium) also caused lysosomal dysfunctions, suggesting an important role of autophagy by nanomaterials (Chen et al., 2014). Autophagic protein LC3 is associated with the toll-like receptor signaling pathway of cells (Acharya et al., 2016). These studies indicated that soots being nanoparticles may affect autophagy mechanism and thus induce cell proliferation. It is noted that cellular accumulation of silica nanoparticles causes lysosomal dysfunctions and co-localizes with the autophagic proteins (Schutz et al., 2016). Surface properties and core stability of nanoparticles were found to be responsible for co-localization with autophagic molecules and trigger the mTOR pathway in hepatocellular cells (Lunova et al., 2017). We observed that fullerene soot particles are internalized and co-localize with the autophagic protein p62, which reflects an important role of autophagy in the trigger of soot particle degradation or digestion (Figure 5B).

**FIGURE 8:**
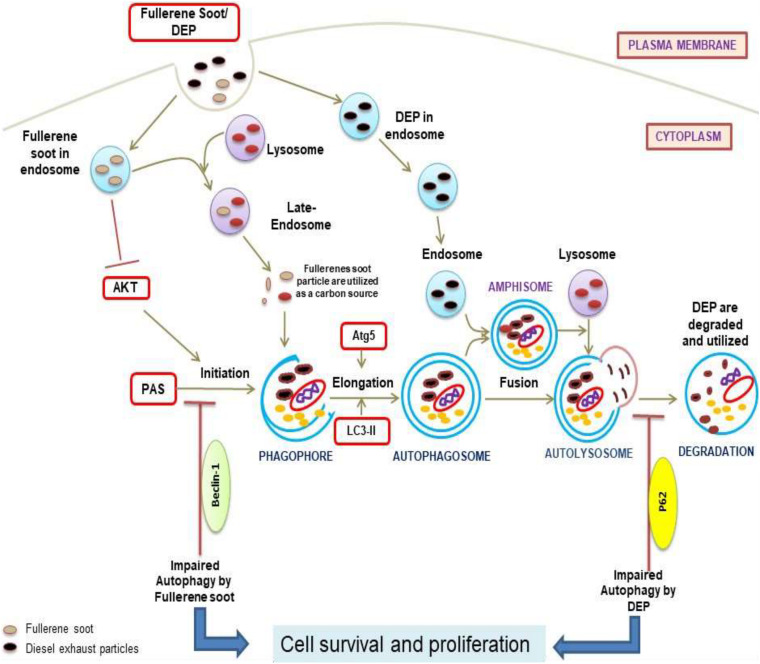
Diagram representation of the proposed mechanisms of action due to soot (fullerene soot and DEP)-mediated inhibitions of autophagy and subsequent induction of cell proliferation. As seen in the figure, fullerene soot inhibited autophagy at the level of beclin as they are directly utilized as an energy source. However, DEP inhibits the autophagic flux and does not allow the autophagy to complete possibly by regulating the expression profile of p62.

To understand the autophagic mechanism of soot-mediated regulation of cell proliferation, we starved A549 cells and triggered starvation-induced autophagy (Huang et al., 2013; Mofarrahi et al., 2013). The PI3/Akt signaling pathway is a key regulatory mechanism involved in autophagic response induced by starvation (Wu et al., 2009; Wang et al., 2013). Akt induction is a survival pathway, and inhibition of Akt is linked with the inhibition of cell proliferation (Bokobza et al., 2014). As depicted in the results, starvation of cells to glucose induces autophagy in A549 cells, which was significantly inhibited by the treatment of fullerene soot. This indicated that fullerene soot might be a potent inhibitor of starvation-induced autophagy response. As shown in Figure 3E, fullerene soot-induced cell proliferation is significantly inhibited by Akt Inhibitor X, suggesting that it is an Akt-dependent pathway. It is important to note that, apart from cell proliferation, Akt Inhibitor X also decreased the expressions of autophagy genes, showing the strong evidence that fullerene soot inhibits induction of autophagy at very initial points (Figure 8). Furthermore, it is evidenced that activation of Akt-mediated autophagy inhibits apoptosis induced by DRAM-mediated mitophagy (Liu et al., 2014). Therefore, it is believed that fullerene soot might have inhibited apoptosis of A549 cells by a similar mechanism. We have also shown that fullerene soot is converted into organic carbon as measured by the OCEC instrument. Here, for the first time, we report that elemental carbon is converted into organic carbon; however, the detailed mechanism is still a subject of further investigation (Figure 7).

Similar to fullerene soot, DEP also induces cell proliferation of lung epithelial cells but did not downregulate autophagic response (Gai et al., 2014). In fact, diesel exhaust particles increased autophagic response in human lung epithelial cells. Recently, it was shown that autophagy inhibition by the Akt inhibitor is linked with inhibition of cell proliferation (Zhao et al., 2016). Impairment of autophagic flux was connected with cell death; however, the autophagic flux-associated effect on cell proliferation of lung epithelial cells is still less clearly known (Masschelein et al., 2014; Yun et al., 2014). Here, we support the fact that lysosomes trap lots of fullerene soot particles, making it unable to receive cargo from other autophagosomes. This may be due to defective fusion of these organelles with lysosomes. As evidenced in the results (Figure 4F), non-degraded p62 reflects the impaired autophagic flux in DEP-treated cells. Based on the results, we can only speculate that lysosomes may be fusion-defective, thus inhibiting autophagy in DEP-treated cells.

Existing literature suggests that there are Akt-dependent and Akt-independent pathways (Kulik and Weber, 1998; Marsh Durban et al., 2013). DEP inhibited autophagic flux, and this might lead to the cell proliferation of airway epithelial cells (Riahi et al., 2016). Interestingly, it was observed that the Akt inhibitor did not downregulate the diesel exhaust particle-induced cell proliferation. Indeed, Akt Inhibitor X further potentiated the DEP-induced cell proliferation. DEP-induced autophagic response was also potentiated by Akt Inhibitor X. Here, it became clear that different autophagic mechanisms of cell proliferation exist in response to DEP (p62 expression up) and in fullerene soot (p62 expression down). These findings clearly show that DEP-induced cell proliferation and autophagic response is entirely different and does not depend on the Akt pathway (Memmott and Dennis, 2009). A previous study described that BRAFV600E, a molecule, coordinates to the PI3K signaling to control melanoma cell proliferation which is independent of AKT signaling and supported the existence of the Akt-independent pathway in the regulation of cell proliferation (Silva et al., 2014). This study also strongly supports our findings, indicating that DEP may be following Akt-independent pathways for the regulation of cell proliferation. Notably, DEP-induced cell proliferation is inhibited by autophagic inhibitor 3-MA and chloroquine but not by COX-2 inhibitor. “Etoricoxib” again pointed toward different mechanisms of autophagy (Kaewjumpol et al., 2018). Chloroquine and 3-MA show inhibition of cell proliferation in a number of cell types, providing support to the present findings (Fan et al., 2006; Jiang et al., 2008; Dong et al., 2019).

## Conclusion

It can be said that autophagy is a key regulatory mechanism responsible for the cell proliferation in response to exposure of soots. The present finding of autophagic mechanism for the control of cell proliferation may throw light on the pathophysiology of tissue remodeling of severe diseases. As the problems of respiratory dysfunctions due to air pollution are increasing throughout the world, proper knowledge about the pathophysiological processes would be a tremendous advantage. Fullerene soot downregulated autophagy and thus is able to cause cell proliferation of lung and other cell types. This cell proliferation (hyperplasia) may initiate the development of tissue remodeling, affecting asthmatic pathology. In this study, the effect of fullerene soot is because of its chemical composition and structural properties rather than its antioxidant effects. Similar to fullerene soot, DEP also caused cell proliferation of cells but did not downregulate autophagic response. These results suggest that more than one type of autophagic mechanism operate in response to different types of soots. Irrespective of encouraging results which may be of clinical relevance, a reassessment of study may be required to further strengthen or revalidate the findings.

## Data Availability Statement

The raw data supporting the conclusions of this article will be made available by the authors, without undue reservation.

## Ethics Statement

The animal study was reviewed and approved by Institutional Animal Ethics Committee, IIT, Kanpur.

## Author Contributions

AKT and SNT conceived the idea. RN performed all experiments in cell culture and mouse models, did the analysis and interpretation of data, and wrote the manuscript. KPM provided his assistance during the experimentations. AKT contributed in critically analyzing the data, reviewing and correcting the manuscript, and funding support. All authors contributed to the article and approved the submitted version.

## Conflict of Interest

The authors declare that the research was conducted in the absence of any commercial or financial relationships that could be construed as a potential conflict of interest.

## Abbreviations

3-MA: 3-methyladenine
AD: aerodynamic diameter
Akt: protein kinase B
AMPK: adenosine monophosphate-activated protein kinase
ATG-5: autophagy protein-5
COX-2: cyclooxygenase-2
DCF-DA: dichloro-dihydro-fluorescein diacetate
DEP: diesel exhaust particles
DMEM: Dulbecco’s Modified Eagle’s Medium
GF: glucose free
GP: glucose plus
HBSS: Hank’s balanced salt solution
HRP: horse radis peroxidase
LC3: light chain 3
LPS: lipo-polysaccharide
OCEC: organic carbon/elemental carbon analyser
p62/SQSTM1: sequestosome 1
PAHs: polycyclic aromatic hydrocarbons
PAS: pre-autophagosomal structure
PM: particulate matter
ROS: reactive oxygen species.
