# Supplementary material for: Proliferation of Lung Epithelial Cells Is Regulated by the Mechanisms of Autophagy Upon Exposure of Soots
Source: Front Cell Dev Biol. 2021 Jul 21;9:662597. doi: 10.3389/fcell.2021.662597 (PMC8335634; doi:10.3389/fcell.2021.662597)
Supplement: Supplementary file 1 [file Data_Sheet_1.PDF]

# Supplementary information

## Results:

### 1. Fullerene soot induced cell proliferation of A549 cells in phosphate buffered saline (PBS).

Fullerene soot increased cell proliferation of A549 cells in absence of regular DMEM medium. In order, to assess the effect of fullerene soot as a carbon source and as cell proliferating agents we have tested its effect on cell proliferation of human lung epithelial cell line at higher concentration (2000 µg/ml).  As seen in the figure, fullerene soot 2000 µg/ml has significantly increased the cell proliferation of human cells in 48 hours.

**Sup Fig. 1.**

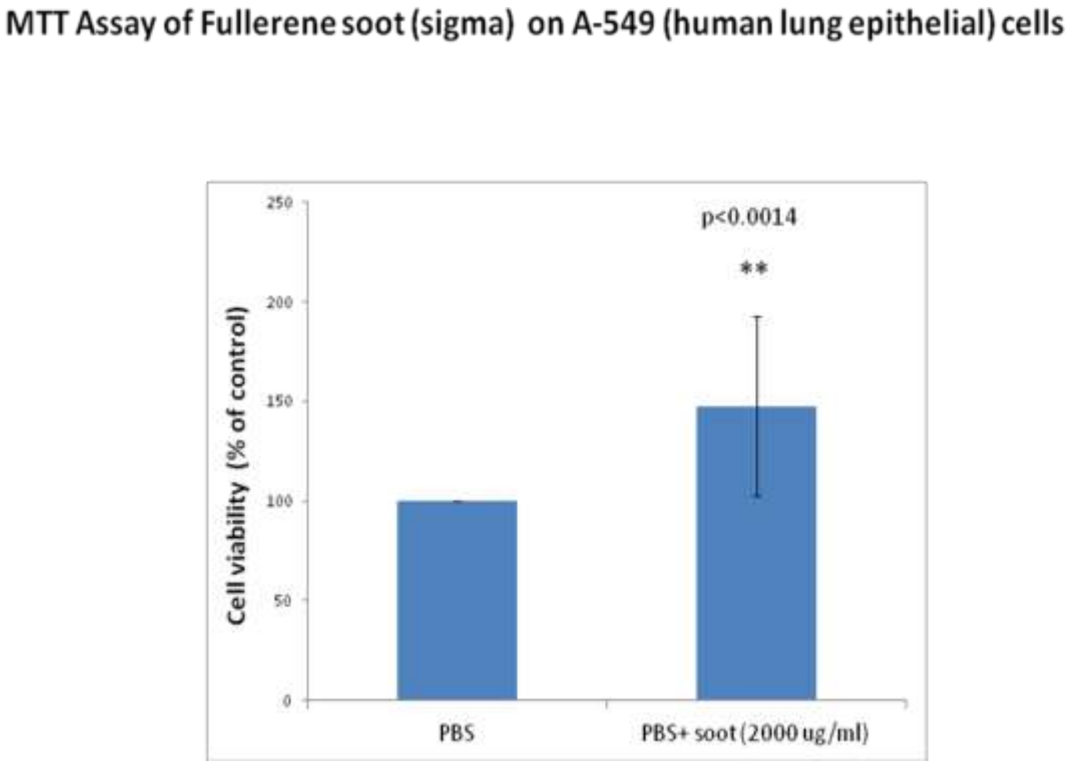


**Supplementary figure 1: Cell proliferation of A549 cells in PBS.** Histograms represent the Mean ± SEM of the different treatment groups.   **=p<0.001 treated versus control.

**2. Fullerene soot inhibits LPS induced LC3 expression in A549 cells.**

It is known that LC3 is the marker for the autophagy. We checked the expression profile of LC3 in response to soot. As shown in the figure LPS has significantly up regulated expression profile of LC3 as seen by the punctate staining in the cells (middle panel). Exposure of fullerene soot has significantly inhibited LPS induced induction of LC3 expression in a 24 hour of exposure period.

**Sup Fig. 2.**

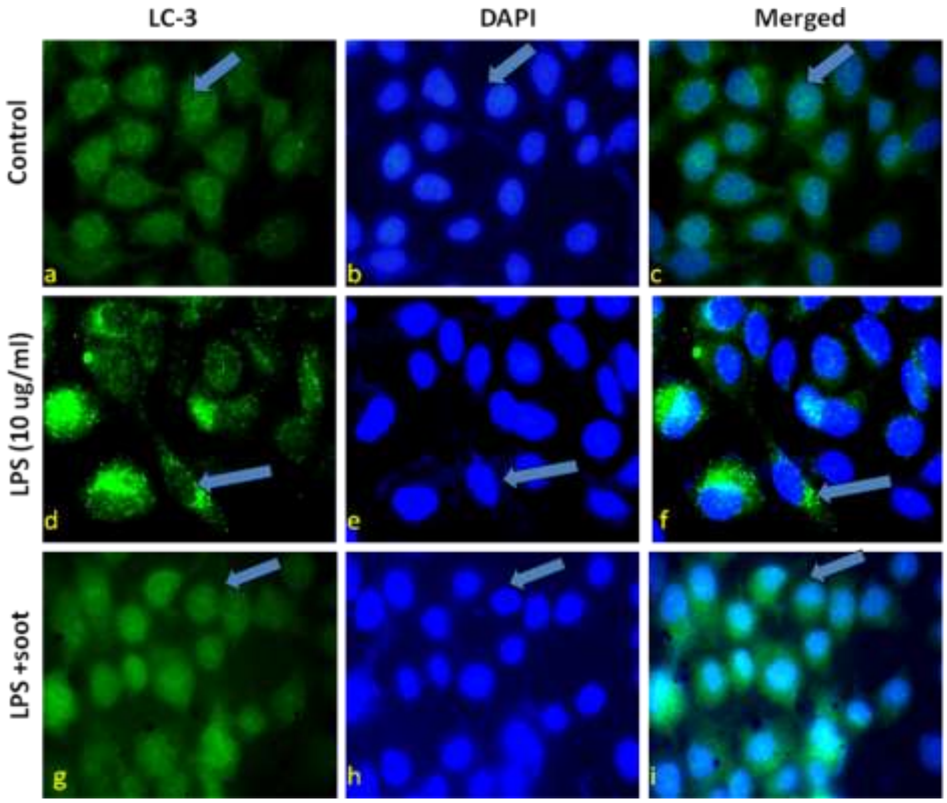


**Supplementary figure 2:** Soot inhibits the LPS induced autophagy in lung epithelial cells. A. LC3 punctate staining in control A549 cells. a- c, immunofluorescence photomicrographs of A549 cells are shown after the 24hrs of control (no LPS) cells. Green fluorescence shows the LC3 punctate staining. panel shows photograph at 100X original modification. d-f, LC3 punctate staining in LPS treated A549 cells. Immunofluorescence photomicrographs of A549 cells are shown after the 24hrs of LPS (10 µg/ml) exposed cells. g-i, LC3 punctate staining in LPS + soot (250 µg/ml) treated A549 cells.

## 3. Effect of LPS alone and in combination of soot on the expression profile of Ki-67 protein in human lung epithelial cells A549.

To assess, whether soot potentiate LPS-induced cell proliferation, we have tested the effects of LPS alone (10 μg/ml) and in combination of fullerene soot (250 μg/ml) on the ki-67 expression profile. The Sup Fig. 3 shows that LPS alone has increased the cell proliferation of A549 cells which was further potentiated by the treated with the fullerene soot. Sup Fig. 3 shows the relative expression (densitometric values) of the Ki-67 protein as measured by the western immunoblotting (blots shown in the Fig. 2) of the

### Sup Fig. 3.

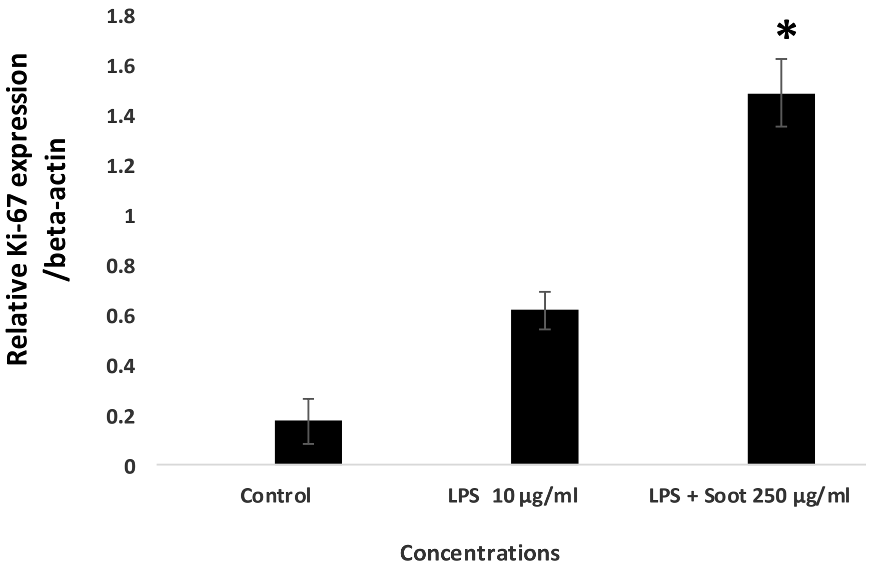


**Supplementary figure 3:** Soot potentiates LPS-induced ki-67 expression in lung epithelial A549, cells. Histograms represent densitometric values of ki-67 proteins as measured by the western immunoblotting. *=p<0.05 compared with control.

**4. Effect of autophagy inhibitor 3-methyl adenine (3-MA) on the DEP induced cell proliferation of A549 cells.**

Further to understand the mechanism of autophagy in response to DEP, we have tested the effects of autophagy inhibiter 3-methyl adenine (3-MA) on A549 cells. Different concentrations (250 µg/ml, 500, µg/ml and 1000 µg/ml) of DEP were exposed to lung A549 cells for a period of 24 hours in the presence and absence of 3MA. As seen in the figure 3-MA significantly decreased DEP induced cell proliferation of A549 cells in dose dependent manner.

**Sup Fig. 4.**

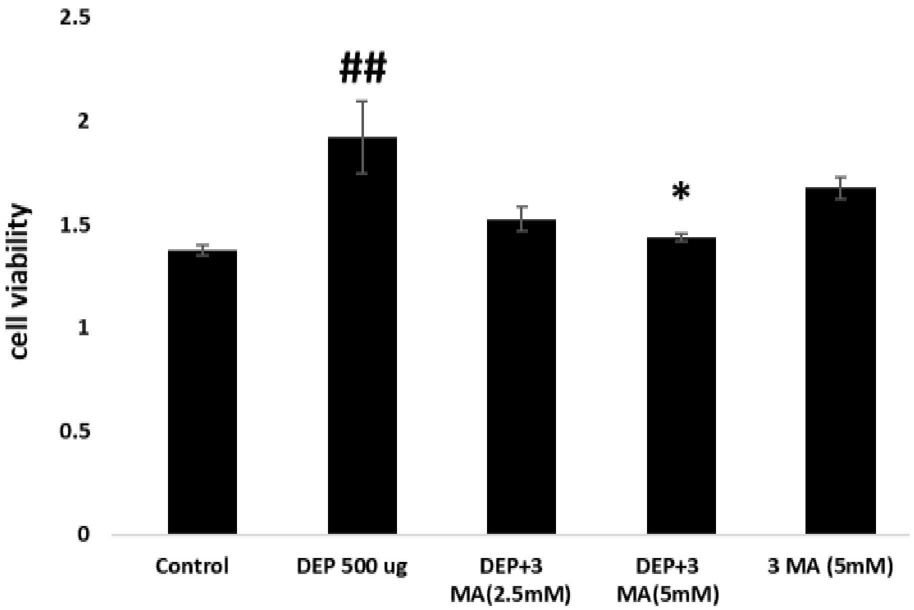


**Supplementary figure 4:** Effect of 3-MA on the DEP induced cell proliferation of A549 cells. Histogram represent relative cell proliferation of A549 cells expressed as Mean ± SE. # #$p<0.01$ compared with control, and * $p<0.05$ compared with DEP treated group.
